# Key challenges in the advancement and industrialization of biobased and biodegradable plastics: a value chain overarching perspective

**DOI:** 10.3389/fbioe.2024.1406278

**Published:** 2024-07-11

**Authors:** Tim Börner, Manfred Zinn

**Affiliations:** ^1^ Institute of Life Sciences, University of Applied Sciences and Arts Western Switzerland (HES-SO Valais-Wallis), Sion, Switzerland; ^2^ Empa—Swiss Federal Laboratories for Material Science and Technology, Technology and Society Laboratory, St. Gallen, Switzerland

**Keywords:** bioplastics, biopolymers, commercialization, sustainability, markets, companies, application, regulation

## Abstract

At the International Symposium on Biodegradable Polymers (ISBP2022) in Sion, Switzerland, experts from academia and industry underscored the remarkable progress in biobased and biodegradable polymers (BBPs) since their initial commercialization around 50 years ago. Despite significant advancements, the technology readiness level (TRL), market adoption, and industrialization of BBPs is not yet competitive to conventional plastics. In this perspective, we summarize the challenges and requirements for advancing the development and industrialization of BBPs, drawing insights from international experts coming from academia and industry, who had participated in the survey and podium discussion during the ISBP2022. In fact, BBPs grapple with persistent and emerging challenges throughout the value chain. These challenges can be grouped into four areas and involve i) the pursuit of sustainable feedstocks together with efficient production and downstream processes as well as recycling technologies and infrastructure; ii) meeting or revisiting product requirements by industry, markets, and consumers; iii) navigating a non-level playing field in their sustainability assessment (LCA) compared to conventional plastics; and iv) struggling with underdeveloped and partially biased policy and financial frameworks as well as lacking clear definitions, terminologies and communication.

## 1 Introduction to biobased and biodegradable polymers (BBPs)

Conventional, fossil-based plastics have historically been designed for durability and toughness affording long lasting products, which are, however, embedded in a linear economy framework. While the non-degradability of conventional plastics is desirable in the production and use phase, it has brought about issues at the end-of-life (EoL), that are the limited recyclability and environmental pollution by persistent micro- and nanoparticles. A significant body of evidence suggests adverse effects of microplastics on humans and animal health, biodiversity, and soil quality, as well as their contribution to climate change (WWFthe Ellen MacArthur Foundation and BCG, 2020; Marfell et al., 2024). Furthermore, global, fossil-based plastic production is responsible for at least 4.5% of greenhouse gas (GHG) emissions (Cabernard et al., 2022).

Consequently, much effort has been invested into the research and development of BBPs to raise their technology readiness level (TRL) as sustainable alternatives to conventional, non-biodegradable plastics. Biodegradability is important for applications having an intended use or likely fate of ending up in the natural and engineered (e.g., wastewater treatment, industrial composting) environments, such as cosmetics and agricultural formulations (e.g., seed coatings and mulch films), protective films for laundry and detergent pods, gras trimmer lines, seedling pods, fishing gear, food packaging, e.g., coffee capsules, tea bags, fruit and vegetable sticker, etc. But also, for products that experience significant abrasion during use, such as shoe soles, tires, artificial turf, protective coatings and paints, etc. (Bertling et al., 2018; Bertling et al., 2021). Biodegradation of BBP-based products at their EoL avoids the formation of persistent microplastics and results in biomass, biogenic CO_2_ or methane (CH_4_). In addition, organic recycling via industrial composting and anaerobic digestion can valorise the biogenic carbon in BBPs in the form of methane and compost for energy recovery, chemical and biogenic feedstock. Organic recycling is particularly useful for post-consumer waste containing significant organic loadings (e.g., food residues), and are thus difficult to recycle mechanically and likely entering and contaminating organic waste streams with non-biodegradable (micro) plastics (European Commission, 2022). Importantly, BBPs are also mechanically and chemically recyclable (Kumar R. et al., 2023). Moreover, the biodegradability of BBPs can be leveraged for creating a circular material and bioeconomy via a technical recycling loop employing biotechnological and chemical recycling (see, e.g., reviews by García-Depraect et al., 2021; von Vacano et al., 2023). Finally, BBPs show on average 20%–30% and up to 40%–50% lower carbon footprints (cradle-to-gate) as compared to conventional, fossil-based plastics (RCI, 2024; Vom Berg and Carus, 2024).

**FIGURE 1:**
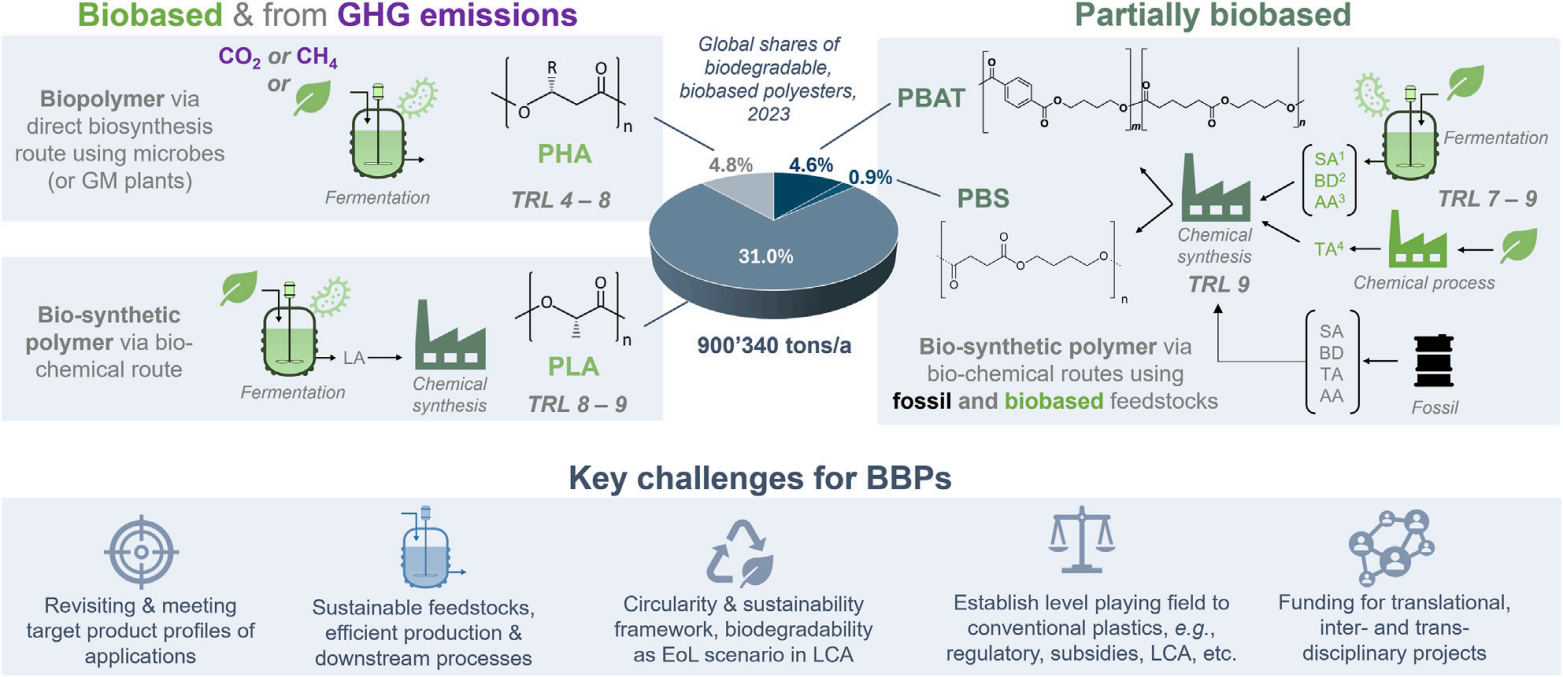
Major biobased and biodegradable polymers (BBPs) with polyester backbone having thermoplastic properties segregated by type of feedstock, manufacturing process (fermentation and chemical) and BBPs production volumes (European Bioplastics, 2023). Production processes at TRL seven to nine contribute to the global BBPs production capacity (2023), lower TRLs are indicative to represent the maturity range. Technology readiness level (TRL) definition according to the European Commission (EC, 2013). Renewable feedstocks from plant biomass, organic waste (TRL 5–6) but also from animal-derived fats (TRL 4, e.g., Gutschmann et al., 2023) are indicated by the green leave icon. Source of TRL for biobased monomer production: ^1^ Roquette Fères, 2024 (FRA), ^2^ Cargill, 2021 (United States), ^3^ Toray Industries, 2022. (JP), ^4^ Origin Materials, 2024 (United States) and Ewing et al., 2022. Abbreviations: Genetically modified, GM; polyhydroxyalkanoates, PHA; polylactic acid, PLA; polybutylene adipate terephthalate, PBAT; polybutylene succinate, PBS; polybutylene succinate adipate, PBSA; succinic acid, SA; 1,4-butanediol, BD; adipic acid, AA; terephthalic acid, TA; lactic acid, LA.

BBPs had created already some significant waves during the 1970ies to 1990ies, “fuelled” by the oil crisis, and then during 2000–2010, due to significant performance improvements in polymer properties and production processes. The biodegradable polyesters PLA (polylactic acid), PHAs (polyhydroxyalkanoates), and PBAT (polybutylene adipate-*co*-terephthalate) have achieved to date the highest market traction and production volumes (Figure 1). While PHA is a natural, microbial biopolyester produced via fermentation (e.g., by Kaneka, Danimer Scientific, BluePHA, RWDC Industries) (Koller and Mukherjee, 2022), production of PLA is achieved via the bio-chemical route employing fermentative lactic acid production with a subsequent chemical polymerization (e.g., by Total-Energies and Corbion, 2022, NatureWorks; Commerer, 2024; Teixeira et al., 2023). PBAT is produced by BASF through chemical synthesis from biobased and petrochemical feedstocks.

Despite their increase in technology readiness level (TRL) together with their environmental as well as circularity advantages, the market adoption of BBPs (excluding polysaccharides) remain at low levels with a share of production capacity of only about 0.2% (ca. 900 t/a) in 2023 compared to the global plastics market (European Bioplastics, 2023). PLA holds the largest share (75%) of the global BBP production volume (Figure 1) and exhibits the highest market adoption with key applications in 3D-printing, FDA-approved medical implants and devices (Tyler et al., 2016), fibres (carpet, textiles), mulch films, food packaging, etc. (Fiori, 2014). Importantly, whilst PLA is only certified for industrial composting conditions, its biocompatibility in humans has been demonstrated *in vivo* for medical implants (Da Silva et al., 2018). While PHAs are widely biodegradable in natural and engineered environments including some medical applications (sutures made of poly (4-hydroxybutyrate)), PBAT biodegrades in soil and under home and industrial composting conditions. Polybutylene succinate (PBS) only biodegrades under industrial composting conditions, whereas its co-polymer with adipic acid (PBSA) shows similar biodegradability to PBAT (for overview see Nova Institute, 2021).

In the last decade BBPs have gained further momentum through the increased environmental awareness of consumers, customers, and industry as well as by policy and governmental bodies. On the other hand, successful applications and industrialization of BBPs are still hindered by several key challenges that require solutions to yield economically viable BBPs and BBPs-based products as sustainable alternatives to conventional plastics. The identification and elaboration of these key challenges in accelerating the development and industrialization of BBPs have been discussed by leading experts from academia and industry during the International Symposium on Biopolymers (ISBP2022) in Sion, Switzerland (see HES-SO YouTube channel, 2022).

## 2 Key challenges for the advancement and industrialization of BBPs

**FIGURE 2:**
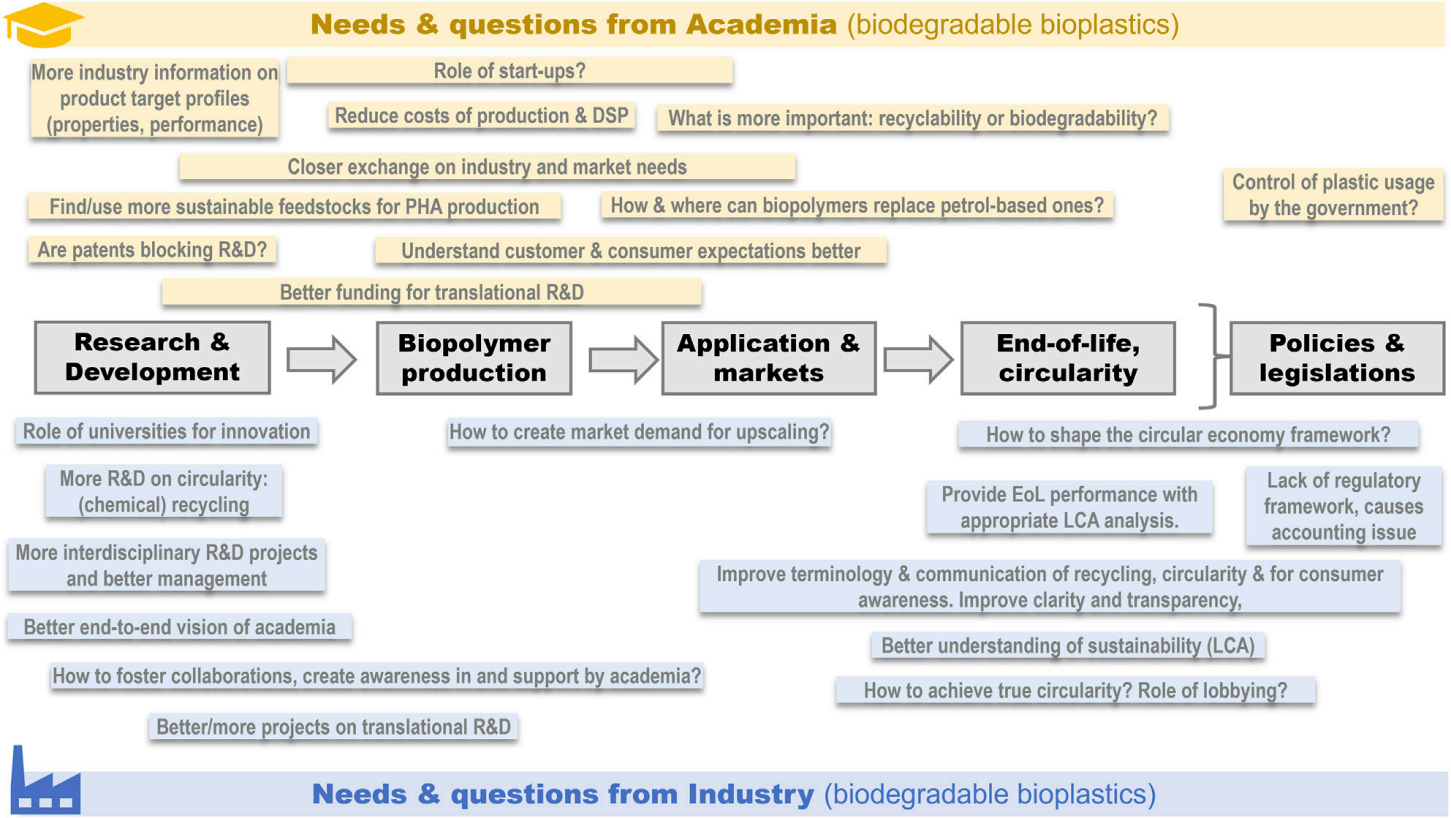
Key challenges identified by academic and industrial experts at the ISBP 2022 in accelerating the development and industrialization of biobased and biodegradable polymers (BBPs) listed across the value chain (grey boxes), including policy, regulatory and legislative framework.

Figure 2 lists the various challenges across the BBP value chain that have been identified by experts during ISBP2022 and will be discussed in more detail as follows.

### 2.1 Meeting the target product properties (TPPs) and managing expectations

One key challenge for BBPs lies in achieving the TPPs demanded by industry, customers, and consumers as well as regulatory bodies. Meeting these TPPs is critical to trigger market demand and market adoption. However, corporates may need to revisit and most likely re-define their product requirements through the lenses of sustainability and with the knowledge of the associated recycling and other EoL challenges. Expectations of corporates are frequently to obtain “drop-in” solutions that are biobased and radically change the EoL properties but maintain all other features of the “conventional” plastics (price, availability, stability, processibility, user performance, etc.). In case of PLA, some successful polymer processing developments for challenging application formats have been made (Fiori, 2014), including foamed PLA as recently reported by Sulzer (Commerer, 2024). Similarly, to enhance the toughness of PBAT, a blend with PLA was shown to yield satisfactory properties for various film applications (e.g., ecovio^®^ by BASF). Similarly, Danimer Scientific is blending PHA with PLA or other short-chain length (scl-) PHAs to improve biodegradability and processibility (Plastics Technology Online, 2024; Commerer, 2021). Developments in formulation, processing and (co-)polymer composition will expand the applicability and product portfolio of BBPs and thereby boosting their market adoption (Park et al., 2024).

While ensuring biodegradation through microorganisms constitutes an important EoL feature, the hydrolytic stability during processing and application is often compromised. Consequently, adaptation and developments in formulations, compounding, and processing conditions are often required. In fact, process-induced degradation can have a significant effect on BBPs, e.g., PLA (Velghe et al., 2023) and PHA (Yeo et al., 2018). Contrary, higher stability requirements during processing and use, negatively affect the biodegradability (for example, see: Mochizuki and Hirami, 1997). For instance, impact modifiers and other additives enforcing the polymer network can impair the accessibility of enzymes and microorganisms and, thereby, the effectiveness of polymer biodegradation (*i.e.,* rate and/or extent). Further research is needed to build knowledge on the structure-function relationship of various PHA types linked to TTPs, e.g., tensile strength, and elongation at break (Choi et al., 2020). Medium-chain-length (mcl-)PHAs have a broader property and application range than scl-PHAs (Li et al., 2016) but only a couple are at pre-commercial scale TRL 6 (Bioplastech, IRL; TerraVerdae, CAN; Bioextrax, SWE). A production platform affording tailored PHAs (e.g., mcl-PHA) at 10—200 g is in dire need for lab-scale testing experiments. Availability of test material at kg scale is key at a second stage, to conduct industrial application trials. A first coordinated step in this direction has been initiated for PHAs by the GO!PHA organization (online sample shop) and few industrial compounders, such as Helian Polymers (NL).

### 2.2 Advancing and scaling of BBP production

#### 2.2.1 Producing BBPs from alternative and more sustainable feedstocks

Whilst PLA, PBAT, and PBS are synthesized via chemical polymerization from their respective (fossil or biobased) monomers, PHAs are produced intracellularly by microorganisms, microalgae, and in transgenic plants (Koller and Mukherjee, 2022). Commercially available PLA and PHAs, as well as the biobased monomers for PBAT and PBS, are produced via microbial fermentation using primarily first-generation feedstocks, i.e., sugars and oils from crops (Figure 1). BBP production processes utilizing more sustainable feedstocks represent another challenge. In case of solid and liquid organic waste streams, the typically heterogenous nature and (seasonal and regional) variations in composition require the development of efficient (and flexible) pre-treatment and fermentation processes for both monomers (Mancini et al., 2020; Bibra et al., 2022; Song et al., 2022) and biopolymers (Molina-Peñate et al., 2022; Kumar V. et al., 2023). Concomitantly, metabolic engineering strategies (Wang et al., 2023) and/or use of microbial mixed cultures (Pagliano et al., 2021) have been developed at lab-scale to enhance the capacity of microbial PHA producers and to synergistically utilize recalcitrant and complex organic waste streams more efficiently.

Adopting and developing process strategies to handle complex and varying feedstock compositions is essential in yielding constant polymer (e.g., PHA) quality as well as appropriate productivity (Wang et al., 2021). Also, establishing cost effective supply chains of novel feedstocks is typically achieved through the economy of scale. Bioprocesses using alternative, more sustainable feedstocks such as organic waste streams and greenhouse gas (GHG) emissions are at a lower maturity level, however, spreading from first proof-of-concept (TRL 3) and small-scale laboratory prototypes (TRL 4) to larger scale pilots (TRL 5 & 6) as well as to demo systems (TRL 7) and first-of-a-kind (FOAK) facility (TRL 8). Commercial scale (TRL 9) is reached only if volumes and quality meet customer demand. (Note, the authors adopted the TRL definition recommended by the European Commission, 2013) Importantly, due to lack of publicly available process data, it remains difficult to conclude on the actual TRL and if such BBPs production processes are operated in an environment of pre-commercial scale (as required for TRL 7) or if manufacturing issues have been solved to reach TRL 8. Notable examples of startup companies demonstrating TRL five to six for the fermentative production of scl-PHA from waste streams are Paques Biomaterials (NL) using municipal wastewater (and sludge), PlantSwitch (United States) and Venvirotech (ESP) using agricultural waste. Newlight Technologies (United States) announced to have advanced to a FOAK in 2019 with thousands of tons of PHA per annum using methane rich emission gases from industrial digestors (Bioplastics Magazine, 2020). In 2015, the company reported a production volume of 45 t/a, a scaling factor of about two orders of magnitude (Bioplastics News, 2015). Mango Materials (United States) employs a similar technology for PHA production from raw biogas and is close to demo system scale (TRL 7) with 5 t/a (Tullo, 2019). All the above companies produce scl-PHAs, which have a limited application range (see Section 2.1).

#### 2.2.2 Fermentation and downstream processes

Fermentation enables the direct biosynthesis of PHAs and monomers for BBP production from various biobased feedstocks (Figure 1), but current BBP production costs are 2–10 times higher as compared to the chemical production of conventional, non-biodegradable plastics. Besides cost reduction through sustainable feedstock supply and pre-treatment (see Section 2.2.1), advancement of fermentation technologies, production strains, and more efficient DSP methods are needed to afford high quality PHAs and monomers at competitive costs. The main process challenges, particularly for PHAs, are summarized below.

• Increase of bioprocess productivity towards the chemical industry’s performance metrics (i.e*.*, 100 g/l/h (Lange, 2021)) by advancing and scaling continuous fermentation, for example, which also affords tailored, high quality PHAs as compared to fed-batch processes (Zinn et al., 2004; Hanik et al., 2019; Dong et al., 2023). Realizing high cell density of 100 g/l in continuous fermentation, which is half of what has been reported for fed-batch (Arikawa et al., 2018), together with a growth rate of 0.5 h^-1^ and 80% PHA content, for example, would yield a >20 fold higher productivity (of 40 g/l/h) compared to the state-of-the-art in continuous PHA production (Koller, 2018). Such bioprocess metrics would also simplify the DSP of PHAs, reduce waste, and enable direct processing of PHA-biomass (see Collet et al., 2022).
• Energy-efficient fermenters with high mass transfer rates (e.g., *k*_L_ *a*), gas recycling, etc. Advance and scale aerobic (hydrogen-oxidizing) fermentation systems (>TRL 5) for direct conversion of CO_2_ (Miyahara et al., 2022; Lambauer et al., 2023) with higher productivity.
• High carbon yields (≥90%) also for alternative feedstocks. C-yield of ∼99% in commercial scl-*co*-mcl-PHA production from palm oil is possible (personal industry communication).
• Optimal DSP methods depend on the type of PHA, and monomer used in BBP production. Reduce DSP cost contribution from ≥50% (Pérez-Rivero et al., 2019; Zytner et al., 2023) to 20%–40% of total production costs (Straathof, 2011). Depending on the methods used, scl-PHA can be recovered and purified today at costs of 1.1—5 €/kg PHA (De Koning et al., 1997; López-Abelairas et al., 2015). Solvent-free recovery of scl-PHA granules (Latex) may be 50% cheaper (Bioextrax, 2023); yet, efficient and sustainable solvent-based DSP methods are typically needed for mcl-PHAs (Hahn et al., 2024).
• Reduce waste and valorise residual cell biomass for additional revenue streams (e.g., Pesante and Frisont, 2023)

### 2.3 More value-chain overarching collaborations through inter- and transdisciplinary projects

To develop and scale technical solutions for the maturation of the BBP value chain (Figure 2), interdisciplinary and transdisciplinary projects are needed, including experts in material and process engineering, environmental sciences, biotechnology, regulatory affairs, marketing, and related fields. While academia often lacks the technical information for polymer processing and application properties (i.e., TPPs), the industry frequently lacks the in-depth research and development capabilities/capacities at the interdisciplinary level. Therefore, translational R&D projects based on pre-competitive collaborations between academia and industry will help to better define technical targets and facilitates communication across the value chain, including policymakers, and consumers.

### 2.4 A none-level playing field for BBPs vs. conventional plastics

#### 2.4.1 Learning curve effect: it takes R&D, capital investments and time

Growing market shares and competitiveness depends on the learning curve (or experience curve) effect, that is, learning-related cost advantages are achieved by companies through increasing R&D and capital investments (Liebermann, 1984; Lieberman, 1989). Interestingly, the initial market demand of PLA was artificially hyped by producers, which then led to disappointment by customers and a drop in demand due to insufficient functionality and performance (Befort, 2021). PLA capacities grew from 70′000 t/a in 2003 to about 459′000 t/a in 2022 with an average market price of about US$1.6—$2.3 per kg (Jem and Tan, 2020; Teixeira et al., 2023). According to the Jem’s law (Jem and Tan, 2020), the PLA demand has been doubling about every 3–4 years since 2007 and is likely to exceed the current production capacity making it less cost competitive to conventional plastics (<1–4 € per kg). This mismatch of demand and supply as well as struggling to meet TPPs is, however, normal for early-stage products, as they require further developments, optimization and growth across the value chain to profit from the learning curve and economy-of-scale effect. PHAs, PBAT and PBS are in a similar learning curve dilemma. In comparison, efficiency gains in oil supply and plastic production resulted in significant cost reduction of 19%–37% per doubling of the polyethylene (PE) production volumes (Simon, 2009). This resulted in market prices of 2.5–4 € per kg in the 1970ies and to around 1 €/kg PE by 2020 (Statista, 2023). A simple comparison reveals that BBPs stand today at a similar product maturity level as PE in 1970ies (not adjusting for currency inflation). Therefore, the challenge for the BBP value chain to become competitive is to either rapidly lower the production costs or to create a framework allowing to accept these costs and/or to temporarily buffer them until sufficient maturity is reached. Besides production costs, the product environmental footprint has gained significance as both policy and economic factor (Damiani et al., 2022). To accelerate the defossilization and transformation of the plastics industry, it is thus imperative to create a funding and policy framework that shortens the learning curve of BBPs by funding of inter- and transdisciplinary R&D as well as sector coupling and scaling projects, enabling the creation of alternative business models together with capital investments, incentives, standards, etc.

#### 2.4.2 Developing a policy framework that promotes innovation and scaling of sustainable technologies and business models is key to achieve defossilization and circularity

Fermentation technologies can help transitioning from a fossil-based, linear industry and society to a circular bioeconomy (Ewing et al., 2022). Despite the many policy developments, such as the European Green Deal, the EUs Circular Economy Action Plan and Plastic Strategy, the Single-Use Plastic (SUP) directive, and the European regulatory framework on Plastic Packaging Waste Regulation (PPWR), there remains still “an urgent need for clarity, predictability, and confidence in Europe and its industrial policy” (The Antwerp Declaration for European Industrial Deal Summit, 2024).

For example, while current LCA methods are sufficiently developed to provide valuable insights into the sustainability and circularity of different products and technologies, continuous improvements in data quality, methodological consistency, and incorporation of more circular economy metrics are necessary to enhance the robustness, comparability, and reliability of LCAs. “Poor data and outdated methods sabotage the decarbonization efforts of the chemical industry” (Oberschelp et al., 2023). Existing LCA studies do not address environmental or human health impacts of neither non-biodegradable nor biodegradable (micro) plastics (Jiao et al., 2024). Biodegradation as EoL in natural and engineered environments is poorly or not quantifiable in current LCAs. Establishing a level-playing field for both BBPs and conventional plastics/polymers (Miller, 2022; Vom Berg and Carus, 2024) is therefore crucial to all stakeholders across the value chain, including policymakers, standardization, and certification bodies. Such advancements in cradle-to-grave and cradle-to-cradle LCAs will also create higher confidence for decision-making processes, avoid green washing, establish coherent terminology as well as transparent communication to consumers and for corporate sustainability reporting, for example,.

Harmonizing definitions, bans, and targets as well as financing mechanisms also at global scale will help to shorten the learning curve effect and to accelerate scaling of plastic pollution and climate change mitigation strategies (WEF World Economic Forum, Insight Report, 2024). According to SUP directive, the current EU definition on natural polymers would ban products made of PBAT, PLA, PBS and modified PHAs, for example, thereby strongly restricting their applications. On the other hand, the PPWR will enforce from 2033 that compostable packaging be mandatory for tea/coffee bags and capsules as well as for sticky labels attached to fruits and vegetables. Also, harmonization of waste recycling targets is needed together with developing the infrastructures to enable collection, sorting and recycling of BBPs present in mixed waste streams, for example. PLA is compatible with existing polyester (PET) waste streams if its fraction stays below 1% (Total-Energies and Corbion, 2022). Therefore, the handling of small BBP waste streams currently hinders market introduction from an EoL point of view and must normally reach sufficient volumes, so that their identification, sorting, and processing is technically and economically feasible (Kumar R. et al., 2023).

Although governmental funding and private investments in clean tech and sustainable products is having its share, subsidies for fossil fuels had a global record high of seven trillion US dollars in 2022 (IMF Blog, 2023). The fossil fuel and oil industry also receive financial compensations (Timperley, 2021). While individual oil and plastics companies have received various subsidies from three million to US$1.8 billion within defined periods till about 2021 (Steenblik, 2021), the bioplastics industry does not receive such incentives as of today and the manufacturers and value chain members must solely carry their financial responsibilities (Green dot Bioplastics, 2024). On the other hand, bioplastics have been listed as “green investments” by the EU’s Taxonomy Climate Delegate Act (European Bioplastics, 2022). Moreover, carbon taxation (ETC) and the EU’s Carbon Border Adjustment Mechanism (CBAM) have the aim to put a fair price on the carbon emitted during the production of carbon intensive goods, which in turn should benefit BBPs having a lower carbon footprint during production. The price of emission allowances in the EU is fluctuating and had decreased from 100 € to less than 60 € per metric ton of CO_2_ (Statista, 2024). The current carbon taxation levels (0.06 € per ton) do not confer a financial advantage, as the significant price gap between conventional plastics and current BBPs (with 20% to one magnitude higher production costs) cannot be bridged.

## 3 Conclusion

Overall, it becomes apparent that the key needs and questions for academia and industry are complementary and spread over the entire BBPs value chain (Figure 2). BBPs are still at the early learning curve towards maturity, facing challenges in accomplishing the fit-for-purpose status, efficient production, purification and processing with proven viability and superior sustainability as well as resolving feedstock utilization and supply chain challenges. BBPs are facing a non-level playing field compared to the well-established oil and plastics industry in terms of underdeveloped LCA methods, policies, funding, and incentives, for example. Consequently, it is essential to consolidate academic and industry efforts in inter- and transdisciplinary projects to overcome these value chain overarching challenges.

## Data Availability

The datasets presented in this study can be found in online repositories. The names of the repository/repositories and accession number(s) can be found in the article/Supplementary Material.
